# Moving Object Detection on a Vehicle Mounted Back-Up Camera

**DOI:** 10.3390/s16010023

**Published:** 2015-12-25

**Authors:** Dong-Sun Kim, Jinsan Kwon

**Affiliations:** Embedded Software Convergence Research Center, Korea Electronics Technology Institute, Saenari-ro 25, Bundang-gu, Seongnam-si, Gyeonggi-do 13509, Korea; jinsan.kwon@keti.re.kr

**Keywords:** moving object detection, mixture of Gaussians, pyramidal Lucas-Kanade optical flow, backup collision intervention

## Abstract

In the detection of moving objects from vision sources one usually assumes that the scene has been captured by stationary cameras. In case of backing up a vehicle, however, the camera mounted on the vehicle moves according to the vehicle’s movement, resulting in ego-motions on the background. This results in mixed motion in the scene, and makes it difficult to distinguish between the target objects and background motions. Without further treatments on the mixed motion, traditional fixed-viewpoint object detection methods will lead to many false-positive detection results. In this paper, we suggest a procedure to be used with the traditional moving object detection methods relaxing the stationary cameras restriction, by introducing additional steps before and after the detection. We also decribe the implementation as a FPGA platform along with the algorithm. The target application of this suggestion is use with a road vehicle’s rear-view camera systems.

## 1. Introduction

Improvements to the function, reliability, and manufacturing process of various sensors making them small, sensitive, and yet strong enough, have made it possible to use those sensors in automobiles [1,2,3,4]. One of its major applications is Backup Collision Intervention (BCI), which informs drivers of obstacles behind the vehicle by giving visual, aural, or tactile feedback. So far this technology has utilized laser, ultrasonic, microwave radar, and vision sensors to measure the distance from the vehicle to obstacles. Although they differs by specific application, those on-board sensors work as the main components for the BCI function independently or in conjunction with others. Drivers of vehicles with extended bodies or carrying oversized loads may have difficulty in seeing behind them directly through the rear-view mirror. As a part of BCI components, on-board monitors connected to a camera mounted on the back of the vehicle provide a visual aid to its user, helping the driver avoid obstacles behind while backing-up. In addition to covering the blind spots, further processing of the video feeds may provide more features such as moving object detection. The processed information then can be integrated back to the original video feed.

Although many other sensors detect stationary obstacles well based on the distance between objects and the vehicle, we focus on moving objects that are beyond the active sensor’s range that might still require the user’s attention while backing up. As a practical approach, we do not assume the obstacles to be of any specific type, therefore, in our suggested method the target objects can range from pedestrians to other vehicles. If available, the detection results may also further processed in combination with the results from other sensors to provide on the screen information in integrated form such as distance, speed, and direction.

Meanwhile, vision-based moving object detection is a major theme in the computer vision arena, and vast number of suggestions has been made. However, many of them assume its viewpoint to be stationary [5,6,7,8], because the movement introduced to its vision source is also reflected on the background of the taken scene. Without proper distinction, the mixed motion between the background and foreground object is hard to separate out. Since our target application is a vision-based BCI system mounted on the back of a vehicle, movement of the viewpoint is inevitable. Therefore, in our suggestion, we first compensate for the background movement, called ego-motion, for later processing using the traditional object detection methods. This is possible because of the unique characteristics of the slow-moving back-up movement, which introduces mild motions across the scene. The uniform vectors found all over the frame then be extracted, and based on those vectors, a rough compensation of the background will be made. The actual object detection routine is then performed using the difference between a frame and the compensated background frame at the time. This paper first examines the previous methods for object detection. Details about the suggested procedure then follow, and we discuss about the implementation of the suggestion as a hardware platform.

## 2. Background Works

In computer vision, finding target objects without additional information from other sensing sources is a challenging job [9]. Despite its ill-posed characteristics, various object detection algorithms having been introduced for vision-based applications such as surveillance cameras, robotics, intelligent systems, and smart devices. Yilmaz *et al.* classified those algorithms into four types according to the completeness of the detected target objects: feature point detectors, image segmentation, supervised learning, and background subtraction [10].

Among other methods, we focus on background subtraction methods to extract the moving objects from image frames. Supervised learning methods, on the other hand, use object classifiers in the form of decision trees or networks [11,12,13]. To build those decision-making networks, analysis of the every type of target object is required. However, due to the fact that any kind of object can obstruct the vehicle’s course, it is impossible to prepare a tree for all types of objects, including unknown ones. Therefore learning-based object detection should not be used in a vehicle backing-up scenario.

In this section, we introduce background subtraction methods first, followed by optical flow which widely finds motion vectors between two consecutive frames. In fact, those vectors are key components for deriving the movement of vision source itself. The optical flow will also be used later in detecting, distinguishing, and finding objects which are moving in the source frames.

### 2.1. Background Subtraction

Background subtraction is a method of separating moving foreground objects from stationary background images. In a video sequence taken from a traffic monitoring camera, for example, vehicles can be separated out from stationary objects such as road markings and traffic signs which are found on the same location over frames. This separation is necessary to transform the video data into traffic information since the moving vehicles are of more interest than the road itself in most cases. To separate the foreground and background objects, building the information about background objects is essential. For this purpose, the background image is kept in various forms during the subtraction process. The actual form differs among methods though, keeping the background information as accurate as possible is critical. Among the background subtraction methods, one common background extraction method is to hold pixels relatively consistent across two or more consequent images, while rejecting other pixels whose value rapidly changes.

Frame differences [5] were one of earliest suggestions of a background subtraction process. This generates a background image by simple frame differences between two consequent images, making the method depend solely on the previous frame. Due to its simplicity, this method has modest computational loads and the background image can be generated with only two frames. The simplicity also makes the background references highly adaptive, resulting in very fast updating of background changes. The major drawback is however that moving foreground objects may easily become part of the background if the object is stopped for more than one frame. The interior pixels of an object also may not be distinguished correctly if the inside has a uniformly distributed intensity value, leaving no difference between the two frames.

Instead of simple subtraction, approximated median [6] keeps an accumulation of approximated pixel values, continuously updating the background image in the result. During the approximation, the generated background converges to a median value of all frames, therefore, background objects which are stationary across several frames may be better imprinted than foreground moving objects which have less opportunity to converge. Although the number of frames used in this convergence process depends on the pixel values of the objects, it is generally much longer than the simple frame difference. Temporarily stopped foreground objects or ones with a plain interior can now be better filtered than with simple frame differences, unless the object becomes part of the background by appearing in the same position longer than the originally occluded background.

Gaussian mixture models [7,8], on the other hand, process the input frames in different ways. Unlike the abovementioned methods, background components are accumulated as terms of Gaussian distribution functions, and the terms do not directly depict a background image at a specific moment. Rather, the Gaussian distribution functions decide whether a pixel from the input image is foreground or background. This statistical determinant is effective for minor changes in the background, such as waving leaves, moving clouds, or raindrops. Furthermore, using mixtures of such functions makes the method multimodal. That is, in the moving clouds example, both clouds and occluded sky should be considered as background. Thus, the distribution must be at least bimodal to classify the clouds and sky as background at the same time.

Recent suggestions on the background subtraction, on the other hand, use sophisticated techniques in combination with those basic ones. Several methods use advanced statistic models to extract backgrounds from moving viewpoints [14,15,16,17], while others suggests the use of neural networks or outlier detection models [18,19]. These subtraction models are further categorized into 17 groups according to their main characteristics [20].

### 2.2. Optical Flow

Rather than background subtraction, another strategy to find moving objects from consequent video frames is by using the optical flow of the frames. According to its definition, optical flow is a motion of apparent movement of brightness patterns in an image, making it sensitive to light sources [21]. Finding the movement can be described as finding the displacement δ which minimizes ε in the following equation:

(1) $$\varepsilon (\delta_{x},\delta_{y})=\sum_{x=u_{x}-w_{x}}^{u_{x}+w_{x}}\sum_{y=u_{y}-w_{y}}^{u_{y}+w_{y}}\left(I_{1}(x,y)-I_{2}(x+\delta_{x},y+\delta_{y})\right)$$

with a given point x, y in an image I. Searching for such a value is performed within a small window w. The window should be introduced because it becomes an aperture problem without the window. An adequate window size provides hints on the direction of vectors from searching through neighbouring points. To find displacements between two consequent frames, various optical flow estimation techniques are introduced [21,22,23,24]. The derived displacements, or flow vectors, contain both the directions and magnitude of pixels, and these vectors are grouped together to form objects. Besides optical flow methods, further classification processes are needed to separate the vector group, in turn which represents the movement of an object, into foreground or background. For this purpose, various statistical grouping algorithms, such as RANSAC and K-means clustering are used [25,26].

Both background subtraction and optical flow methods have been widely used for applications including intrusion or motion detection from video stream with stationary cameras. Recent studies showed that the use of grouping algorithms with optical flow also alleviates the limitation of using stationary cameras for some level by further classification of feature points into two different motion groups [25,26]. Rather using classification methods, in our suggestion, we combine a background subtraction and an optical flow process to eliminate the stationary restriction and derive backgrounds at the same time. The key benefits of using this combinational approach are that the background motion, known as ego-motion, can be cancelled by its optical flow vectors, and the each detection result from optical flow and background subtraction can be cross-checked later.

## 3. Moving Object Detection

**Figure 1:**
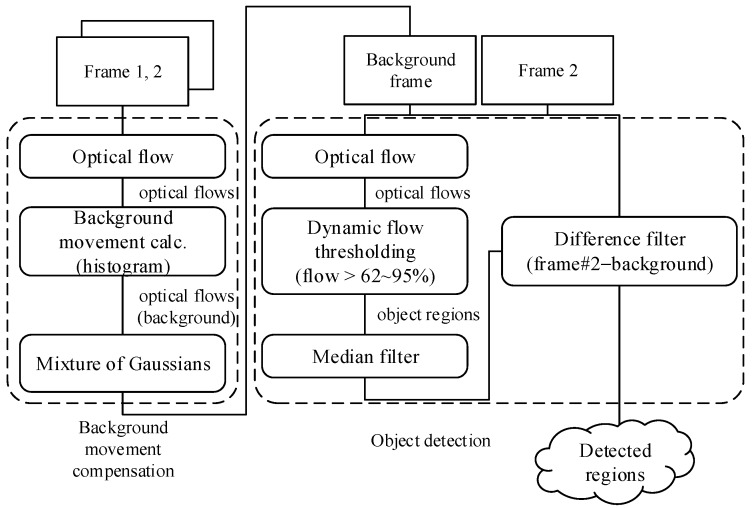
Overall detection procedure of the proposed scheme.

In this section, we present the proposed procedure for moving object detection. To achieve better detection and less false positives than previous standalone approaches with moving view sources, our proposed scheme uses both background subtraction and optical flow methods simultaneously. As shown in the Figure 1, this combinational scheme is composed with the following two step approach: background motion compensation, and object detection. The actual detection of moving objects is done in the object detection part by using the optical flow between a frame and the derived background at the moment. In our suggestion, a background frame is defined by the difference between two video frames, thus, object detection can be started with a sequence having at least two frames.

### 3.1. Background Motion Compensation 

As the camera on the vehicle moves freely, the image sequences obtained from the camera contain motions of both background and target objects. Therefore, when a new frame arrives, the ego-motion of background at that time needs to be eliminated first. In our scheme, the ego-motion is induced by optical flow estimation between the newly captured frame and previously derived background components. The background components, on the other hand, are derived by Gaussian mixture models. Since we have movements in the background, the multimodal distribution functions discussed previously are needed for distinguishing the moving background objects. In this approach, background frames are stored as Gaussian mixture function terms, and if the ego-motion vector is induced, the entire background terms can be shifted back along the vector, resulting in compensated background terms.

**Figure 2:**
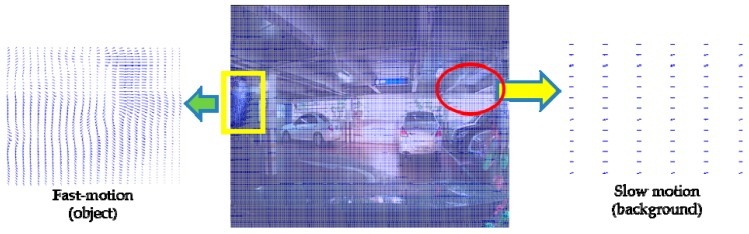
Optical flow between two consecutive frames. In this result, the vectors representing background motions stay consistent while moving object region shows major changes.

**Figure 3:**
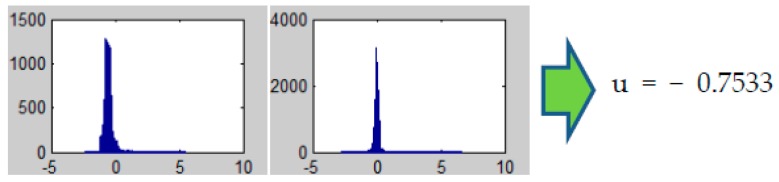
Histograms on the optical flow results. We assume the average value of the most frequent section found on the histogram as the ego-motion.

To derive the motion vector disparities of each pixel, we use an optical flow method which is based on the pyramidal Lucas-Kanade optical flow and warp theory [23,27]. In this method, the overall optical flow is derived through several scales. The input images are scaled down into specified sizes for each level, similar to the pyramid structure. Optical flow results on each level are then scaled back and summed to form the overall disparities for all pixels between the two frames like one in the Figure 2. After the disparity between previous background and current frame has been derived, it is possible to extract the ego-motion from the entire pixel flow. This normalization process incorporates the generation of histogram statistics on all displacements and selection of the most frequent flow as the representative background movement. In our suggestion, the size of each histogram section is adjusted so there exist 100 sections. The estimated ego-motion vector is composed with the average values of the elected sections on each axis, such as −0.75 and −0.02 in Figure 3.

Before compensating the ego-motion, the background component should be built first. There are several methods to do this, as we discussed in the previous section, but we use Gaussian mixture models for their better adaptation to partial movements of the background itself. In this model, pixels of the background frame are stored as mean terms for the Gaussian functions. The mean and deviation terms are affected by upcoming frames with predefined update factors, therefore, the mean term can be changed over frames. At the very beginning of background subtraction, the mean term is initialized by the first frame directly, assuming it is a background image.

With the estimated vector and the subtracted background components, now it is possible to compensate the background motion. This action is done by a simple shift of the mean and deviation terms of the Gaussian Mixture functions along with the estimated vector, but being aware that the estimated movement is usually less than one, so the shift action should be handled as a convolution between the terms and the motion vector. The mean term after the shift action now can be seen as the compensated background.

### 3.2. Object Detection

Although it may seem possible to extract target objects using the frame differences between the compensated background and the current frame, the simple comparison between those frames contains both false-positives and false-negatives. This leads the detection results to have way too much noise and yet even less detection tendency than the results obtained sing optical flow methods alone. To solve this problem, we use optical flow between the background and the current frame to deduce the motion vectors of all pixels. At this point, the resulting vectors which have different magnitude and direction than the background can be grouped into moving regions, and the regions can be reported as the moving objects.

To group the regions from scattered motion vectors, a threshold magnitude value should be presented. In our suggestion, we added a routine that chooses the threshold which can bisect and holds at most 5%–38% of overall vectors in a frame. This is because the optical flow between a background and a frame with relatively big ego-motion tends to yield many more false-positives. In this case, the threshold magnitude needs to be big enough to be less sensitive to both the background and foreground movement, resulting in reduced false-positives. The background motion magnitude determinant has to be chosen based on its application, so that the routine can choose the percentage proportionally according to the determinants.

**Figure 4:**
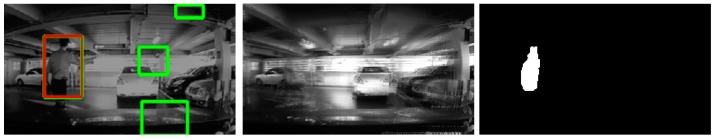
Derived frame difference (**right**) for false-positive filtering. The difference is derived between a frame (**left**) and its background (**middle**) with adequate threshold.

Before reporting the final detection results, it is also possible to further apply a simple filtering process which can reduce false-positives. Since we derived the detection results solely from the optical flow method, the aforementioned frame difference between the background and the current frame is still valid for cross-checking with the results. In this usage, the subset of optical flow results, shown as red and green rectangles in the left picture of Figure 4, can be reported as moving objects which also have been confirmed as moving objects on the frame difference results. Other filtering methods including a median filter on the results are also possible.

## 4. Experimental Results

To evaluate our suggestion and compare the results with other methods, we present two experimental results in this section. In most cases, evaluations on optical flow based methods are usually focused on the terms of precision and recall, relative distance, angular error, and computation time. Since the accuracy is what matters in our target application, we mainly state the precision and recall terms. Precision and recall are defined as follows:

(2) $$\text{Precision}=\frac{\text{True positive}}{\text{True positive}+\text{False positive}}$$

(3) $$\text{Recall}=\frac{\text{True positive}}{\text{True positive}+\text{False negative}}$$

where true positive is the region first detected by the algorithm that turned out to be actually moving. False positive is the region reported by the algorithm as moving when it actually only contains background. False negative is the region which should be reported as a moving object but not detected by the algorithm. Thus, the precision term reflects the accuracy of overall detected results while recall depicts the sensitivity of the algorithm.

**Figure 5:**
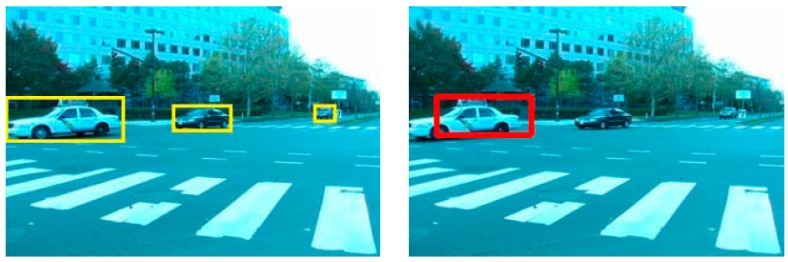
Car example with 200 consecutive frame sets. The rank-constraint models give better results in terms of recall (**left**), however, the precision of overall results is better with the suggested method (**right**) due to the additional filters.

The first sample sequence in Figure 5 consists of 200 consecutive frames capturing three vehicles moving from right to left [28]. The camera also moves right to left along with the first vehicle, leaving ego-motion on its background pixels. In this sample, two rank-constraint models [18,26] result precision of 0.83, 0.95 and recall of 0.99, 0.92 are used, respectively, while the suggested procedure gives precision of 0.98 and recall of 0.78 (see in Table 1). Since the scenario of the target application focuses on rather closer obstacles, better precision on those can draw the immediate attention of the drivers by reducing false-alarms.

**Table 1:** Precision and recall comparison

Algorithm	Precision	Recall
Rank-constraint 1 [26]	0.83	0.99
Rank-constraint 2 [18]	0.95	0.92
Suggested method	0.98	0.78

**Figure 6:**
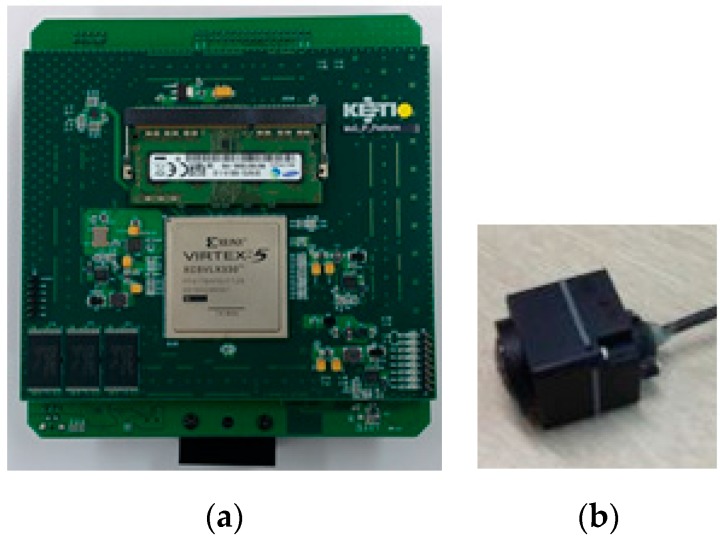
The FPGA platform with camera. The (**a**) platform takes LVDS signal from (**b**) camera and outputs as HDMI signal. External HDMI recorder was needed for further analysis as the platform processes the input frames on-the-fly.

To evaluate the proposed scheme in everyday use, we implemented the scheme on a FPGA platform [29,30] and mounted it on a test vehicle. The platform consists of a camera with resolution of 800 × 480 pixels and a HDMI recorder for further analysis (Figure 6).

**Figure 7:**
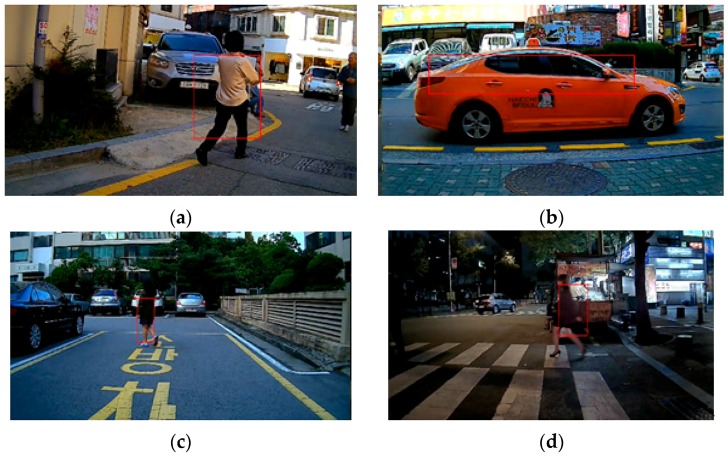
Part of detection results from the test vehicle. Tests were conducted in several places and under various conditions such as (**b**,**c**) parking lots and (**a**,**d**), streets ranging from (**a**,**c**) daytime to (**d**) dusk. All 19 videos including the above four were taken while the vehicle was in backwards motion.

For the test, it took 19 videos 150 frames each, at seven frames per second, having both ego-motion towards the rear side and the target object’s movement. Figure 7 and Table 2 show parts of the sequences with false-positive and negative analysis results on all 19 test scenarios.

**Table 2:** False positive and negative results on the all 19 videos (150 frames each).

**Scenarios**	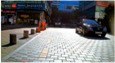	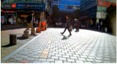	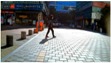	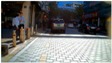	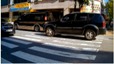
**False-Positives**	0	2	2	0	3
**False-Negatives**	0	0	1	4	0
**Scenarios**	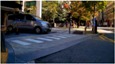	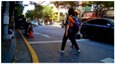	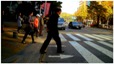	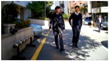	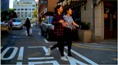
**False-Positives**	1	0	2	0	1
**False-Negatives**	1	0	0	0	1
**Scenarios**	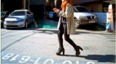	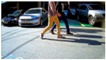	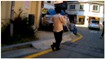	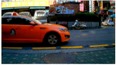	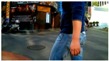
**False-Positives**	0	0	0	1	0
**False-Negatives**	0	1	2	0	0
**Scenarios**	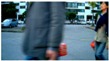	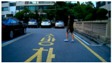	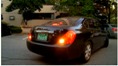	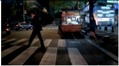	
**False-Positives**	0	0	0	0	
**False-Negatives**	0	1	0	0	

## 5. Conclusions

In this paper, we discussed two types of methods for moving object detection and their constraints. We suggested a combinational method and false-positive filters to relieve the stationary viewpoint constraint. Experimental results show the overall improvement in the term of precision, which is an important feature for use with BCI applications.
